# Newborn screening for central congenital hypothyroidism: past, present and future

**DOI:** 10.1530/ETJ-24-0329

**Published:** 2025-02-19

**Authors:** Mark R Garrelfs, Christiaan F Mooij, Anita Boelen, A S Paul van Trotsenburg, Nitash Zwaveling-Soonawala

**Affiliations:** ^1^ Department of Pediatric Endocrinology, Emma Children’s Hospital, Amsterdam University Medical Centers, University of Amsterdam and Vrije Universiteit, European Reference Network on Rare Endocrine Conditions (Endo-ERN), Amsterdam, The Netherlands; ^2^ Amsterdam Gastroenterology Endocrinology Metabolism, Amsterdam, The Netherlands; ^3^ Endocrine Laboratory, Department of Laboratory Medicine, Amsterdam UMC, University of Amsterdam, Amsterdam, The Netherlands

**Keywords:** central congenital hypothyroidism, newborn screening, multiple pituitary hormone deficiencies, thyroxine, pituitary

## Abstract

Congenital hypothyroidism (CH) is defined as thyroid hormone deficiency at birth and constitutes one of the most common causes of preventable intellectual disability worldwide. Central CH is caused by insufficient pituitary or hypothalamic control of thyroid function, biochemically characterized by a low serum free thyroxine (fT4), in combination with a low, normal or mildly elevated thyroid-stimulating hormone (TSH). Central CH is less common than primary CH and is part of multiple pituitary hormone deficiencies (MPHD) in most of the cases. MPHD at birth, also known as ‘congenital hypopituitarism’, is a potentially life-threatening condition due to the possible co-occurrence of adrenocorticotropin hormone and growth hormone deficiency that can result in severe hypoglycemia and adrenal crisis. To date, central CH is the only pituitary hormone deficiency suitable for newborn screening (NBS), providing an opportunity for early detection of MPHD. Even though the first NBS programs utilized T4-based methods that were able to identify central CH, most countries have since transitioned to TSH-based approaches due to the high rate of false positives associated with T4-based strategies. Now, 50 years after the introduction of NBS for CH, only a few countries around the world have a screening program capable of detecting central CH. In this paper, we review the past, present and future of NBS for central CH. We will outline the importance of early detection of central CH and discuss the challenges and opportunities of screening for this condition.

## Introduction

Congenital hypothyroidism (CH) is defined as a thyroid hormone deficiency at birth and constitutes one of the most common causes of preventable intellectual disability worldwide (1). Early initiation of treatment with synthetic thyroid hormone (levothyroxine) can obviate most neurologic deficiencies, which was the driving factor behind the development and implementation of newborn screening (NBS) programs in the 1970s (2, 3).

Central CH is caused by insufficient hypothalamic or pituitary control of thyroid function, while primary (thyroidal) CH is caused by abnormalities of the thyroid gland (4, 5, 6). Central CH is biochemically characterized by low serum free thyroxine (fT4) in combination with low, normal or slightly elevated thyroid-stimulating hormone (TSH) (6). With an estimated incidence of 1 in 13,000–30,000, central CH is less common than primary CH (incidence 1 in 2000–3000) (6). In most of the cases, central CH is a part of multiple pituitary hormone deficiencies (MPHD), but it can also occur as an isolated condition (6, 7, 8).

**Figure 1:**
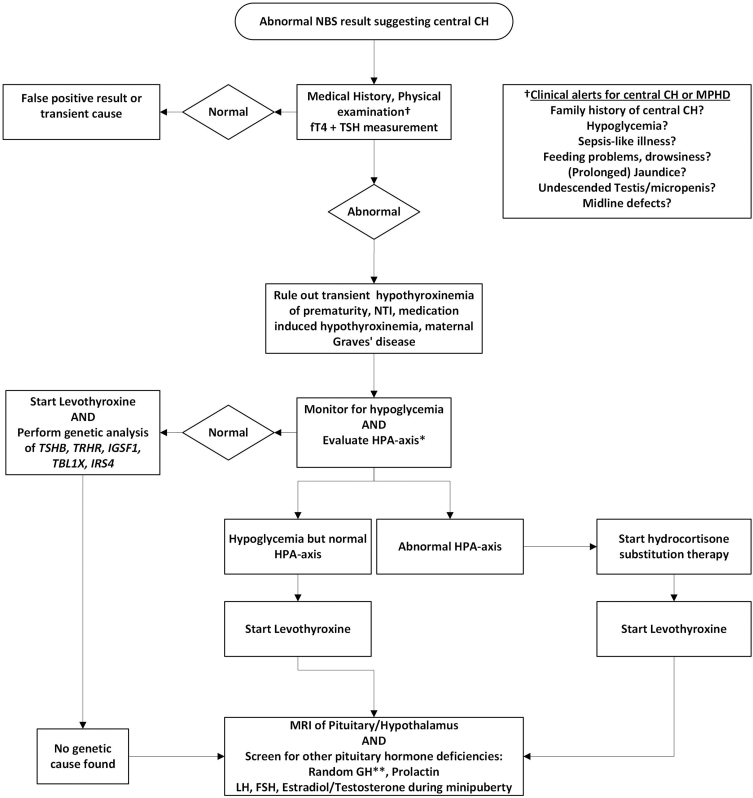
Flow diagram representing the proposed work-up of neonates with an abnormal NBS result suggesting central congenital hypothyroidism. Abbreviations: HPA axis, hypothalamus–pituitary–adrenal axis; NTI, non-thyroidal illness. *We recommend performing a low-dose ACTH test (1 μg) (6). **It is advised to perform several random serum growth hormone measurements in the first month of life. Growth hormone values >30 mIU/L effectively exclude growth hormone deficiency in this age-group (101, 102).

Because (isolated) central CH is often clinically silent, the clinical presentation of patients is mainly dictated by other coexisting pituitary hormone deficiencies (5, 6, 7). Central CH as a part of MPHD or ‘congenital hypopituitarism’ is a potentially life-threatening condition due to the possible co-occurrence of adrenocorticotropin hormone (ACTH) deficiency and/or growth hormone deficiency (GHD) that can result in severe neonatal hypoglycemia and adrenal crisis (9). Even in the presence of MPHD, the clinical diagnosis is frequently delayed by months to years (7). Central CH is currently the only pituitary hormone deficiency detected by NBS, and in countries with a NBS program able to detect central CH, an abnormal screening result is often the first clue to diagnosing MPHD (7). The proposed work-up of a child with an abnormal NBS result suggesting central CH is summarized in Fig. 1.

MPHD is often associated with pituitary malformations, of which the pituitary stalk interruption syndrome (PSIS) is the most frequent and belongs to the spectrum of midline defects (6, 10, 11). It is a phenotypically heterogeneous disorder of complex multifactorial origin (12, 13, 14, 15, 16, 17, 18). A major genetic contribution is suspected, although monogenic causes are extremely rare (12, 13, 14, 15, 16, 17, 18).

In contrast, isolated central CH is mostly a monogenic disorder with a high yield of genetic testing (7). Currently, five genes (*TSHB*, *TRHR*, *IGSF1*, *TBL1X* and *IRS4*) related to isolated central CH have been identified (6, 19, 20, 21, 22, 23, 24). *IGSF1*, *TBL1X* and *IRS4* are inherited in an X-linked manner (6).

An often-overlooked category of isolated central CH are children whose mothers had uncontrolled thyrotoxicosis during pregnancy. These children can be born with a rare form of (transient) central CH, caused by hypothalamus–pituitary–thyroid (HPT) axis suppression *in utero* (25, 26). The medical history of the mother, thyroid functions tests and thyrotropin receptor antibody titer measurement will generally lead to the right diagnosis.

Irrespective of the cause, early recognition and treatment of central CH is essential for improving long-term health outcomes. Even though the first NBS programs utilized a total T4-based approach, most countries have switched to a TSH-based NBS (3). TSH-based screening effectively detects primary CH but misses central CH (3, 27). In this review the past, present and future of NBS for central CH will be discussed. By reporting the benefits and pointing out specific pitfalls, we hope to increase the worldwide incentive to consider incorporating this condition in NBS programs.

## Why NBS for central CH is important

Historically, central CH was thought to be a very rare and mild condition, leading to a lower perceived urgency for its inclusion in NBS programs. However, these assumptions have since been disproven. First, although still classified as a rare disease, central CH is more common than previously thought (28). In countries with longstanding NBS programs that detect central CH, such as Japan and the Netherlands, the incidence is reported to be between 1 in 13,000 and 1 in 16,000 (6), respectively. This is comparable to the incidence of phenylketonuria (PKU), the first metabolic disorder included in NBS programs (29). Second, the presumed mild character of central CH was refuted by a Dutch study of 143 early detected central CH patients (30). Based on initial serum fT4 values, over fifty percent of patients with central CH were classified as having moderate-to-severe CH (fT4 <10 pmol/L or 0.78 ng/dL) (4, 6, 30). Last, the severity of central CH extends beyond hypothyroidism, as between 61 and 98% of patients are reported to have additional pituitary hormone deficiencies (7, 8, 31, 32, 33). MPHD is a potentially fatal condition and central CH is currently the only pituitary hormone deficiency suitable for NBS (7). Screening for central CH thus provides an opportunity for early detection of MPHD. This is important because the general notion that MPHD is usually diagnosed soon after birth based on clinical symptoms is not supported by empirical evidence, and in the absence of screening, the diagnosis is frequently delayed by months to years (6, 7, 31, 32, 34). In a recent study on clinical characteristics of Dutch central CH patients, most neonates with central CH as a part of MPHD were diagnosed only after notification of an abnormal NBS result, even though the majority was hospitalized in the first weeks of life because of feeding problems, hypoglycemia or (prolonged) jaundice (7).

The benefits of early treatment of patients with moderate-to-severe primary CH have been clearly established and this most likely carries over to central CH (35, 36). Unfortunately, in analogy to primary CH, randomized-clinical trials comparing early versus late started treatment have not been performed. There is, however, growing evidence suggesting a significant impact of undiagnosed and untreated central CH on neurodevelopmental outcomes (30, 31, 32, 37, 38). In 2020, Nebesio *et al.* reported that developmental delay was present in 51% of 42 patients with late detected central CH (31). In 2020, Naafs *et al.* performed a systematic review and meta-analysis of available literature and found that full-scale intelligence quotient (FSIQ) scores below 70 (≥2 SD below the norm) were significantly more prevalent among patients with central CH compared to the general population (37). Recently, German *et al.* reported the characteristics of 94 central CH patients identified between 1987 and 2021 and found neurologic sequelae in 37% of patients (32). These negative effects could not exclusively be explained by the coexistence of MPHD as a higher prevalence of neurological sequelae was found in the isolated central CH group versus the MPHD group (60 vs 32%) (32).

Indirect evidence for a positive effect of early treatment on neurodevelopment was shown by Naafs *et al.* in a study comparing early treated central CH patients (detected by NBS) with their healthy siblings (39). In this cross-sectional study, FSIQ was measured in 87 patients (52 with MPHD and 35 with isolated central CH) and compared with 52 unaffected siblings. MPHD patients had a lower FSIQ compared to unaffected siblings (mean difference: −7.9 points, 95% CI: −13.4 to −2.5), while FSIQ scores of isolated central CH patients and siblings were similar (39). This suggests that the direct negative effects of hypothyroidism on the brain in patients with central CH can be prevented by early treatment, similar to primary CH (40, 41).

By logic, levothyroxine treatment cannot negate the potential additional negative effects on brain development caused by hypoglycemia due to ACTH deficiency and/or GHD, but the damage can be reduced by early detection and treatment of these conditions when clinicians are alerted by an abnormal NBS result for central CH.

## History of NBS for (central) CH

The first NBS program for CH was established in Quebec, Canada, in April 1974 (42). This program initially utilized a radioimmunoassay to measure total T4 in dried blood spots (43). Shortly thereafter, a TSH assay for filter paper blood was developed by the same group to speed up detection of newborns with primary CH (42, 44). Although the T4-based approach (either total T4-reflexTSH or simultaneous total T4 and TSH measurement) was widely adopted following recommendations from the American Thyroid Association, acknowledging the benefits of screening for both primary and central CH, it had limitations (42, 45). Low total T4 levels are not able to differentiate between central CH and other benign or transient causes of low T4 such as thyroxine-binding globulin (TBG) deficiency, non-thyroidal illness (NTI) and transient hypothyroxinemia of prematurity (THOP), resulting in false positive screening results (46). The time between blood collection and the moment of diagnosis was rather long and there were some concerns about missing cases of thyroid dysgenesis with borderline T4 concentrations (47). After improvement of the TSH assays, most countries therefore transitioned to a TSH-based screening approach, which effectively detects primary CH (with fewer false-positives and shorter time to diagnosis), but fails to identify central CH (48).

Like several other countries/regions, the Dutch screening program maintained a T4-based approach and refined the screening algorithm overtime with the goals of speeding up the screening process and reducing the number of false-positives. At the start of the Dutch program in 1981, total T4 was measured followed by TSH in those with total T4 concentrations belonging to the lowest 20% of the daily mean (≤−0.8 standard deviation score). In 1982, a decision rule was added to refer premature newborns (birth weight ≤2500 g and a gestational age of ≤36 weeks) only in case of an elevated TSH, reducing the number of false-positive NBS results due to hypothyroxinemia of prematurity (27). To address the issue of false-positives related to (partial) TBG deficiency, TBG measurements (for neonates with total T4 levels in the lowest 5%) were integrated into the screening process in 1995 (49). This three-step total T4-reflex TSH-reflex TBG strategy effectively reduced the number of false-positives while maintaining a positive predictive value for primary CH similar to TSH-based NBS programs, with the advantage of detecting central CH (46, 50).

## Challenges when screening for central CH

Screening for central CH is complicated because it relies on a single biomarker (T4) that is influenced by several factors including binding proteins, the clinical condition of the patient, gestational age and the individual setpoint of the HPT axis.

The biggest challenge when screening for central CH with total T4-based methods is arguably the high number of false-positives caused by TBG deficiency (50). Different strategies have been employed to overcome this problem. In the Netherlands, measurement of TBG is added and a T4/TBG ratio is calculated as a surrogate of fT4 (51). In Japan, the influence of binding proteins is largely negated by directly measuring fT4 in dried blood spots (52). However, low TBG values can still lead to falsely decreased free T4 values (52, 53). For this reason, a fT4 approach does not seem to be superior to a T4/TBG approach (54).

Another significant source of false-positive results in T4-based NBS comes from other low-T4 conditions, such as NTI (50, 55). Thought to be an adaptive response to stress to save energy and protect the body from hypercatabolism, NTI describes the serum thyroid hormone concentration changes that can occur during severe illness (56, 57). It is characterized by low T4, low triiodothyronine (T3) and non-elevated TSH, resembling central CH (56). Although it was once thought that increased serum reverse T3 (rT3) levels were able to discriminate NTI from central hypothyroidism, it is now known that serum rT3 may be either increased, normal or even reduced in NTI patients (56). Nevertheless, measuring rT3 in difficult cases can still prove useful as a high rT3 level is suggestive of NTI (58). NTI is generally self-limiting, with normalization of thyroid hormone concentrations on clinical recovery of the patient, ultimately discriminating NTI from central CH.

Screening preterm neonates with T4-based methods can also generate false-positive results as they often have low T4 in combination with non-elevated TSH levels (59, 60). Factors including loss of maternal thyroid hormone supply, immaturity of the HPT axis with a blunted TSH surge after birth, decreased binding proteins (TBG), medication use and NTI all contribute to the well-recognized phenomenon THOP (55, 61). THOP is the most frequent cause of low T4 levels in premature newborns (62). Thyroid hormone levels are proportionate to the degree of prematurity and tend to normalize when a gestational age of 37 weeks is reached (63).

Of note, clinicians should be aware of the possibility of masked primary CH due to the inability of sick and preterm babies to generate an adequate TSH response in the first weeks of life, warranting post-screening strategies in these populations, as recommended by the American Association of Pediatrics (4, 5, 64).

**Figure 2:**
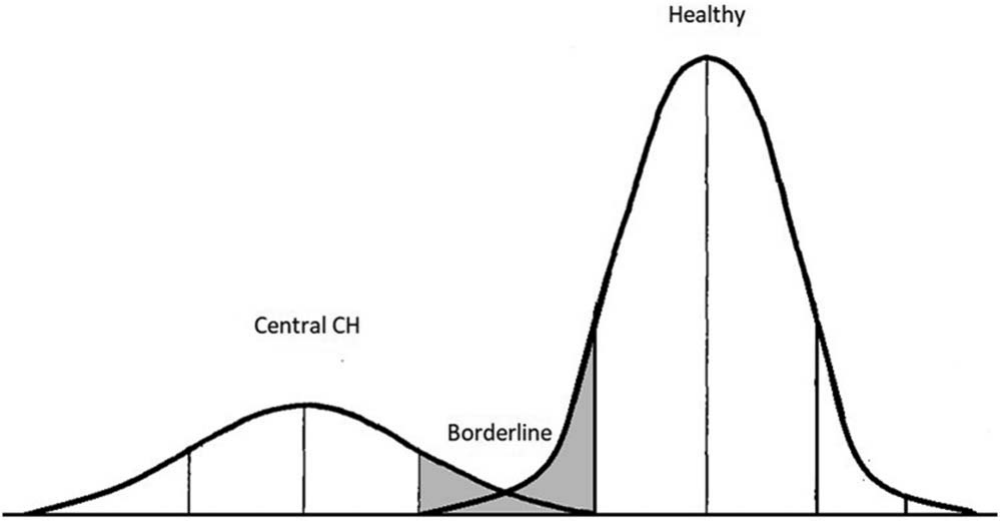
Schematic representation of the normal distribution of total thyroxine (T4). Schematic representation of the normal distribution of total thyroxine (T4) on the horizontal axis, showing the overlap (borderline) between healthy subjects and patients with central CH.

A challenge shared by other conditions in NBS programs is the selection of appropriate cutoff values. Variability in T4 levels across a population is determined by the individual setpoint of the HPT axis. The individual setpoint of the HPT axis follows a normal distribution that causes an area of overlap between healthy subjects, with T4 in the lower part of the Gaussian distribution (false-positives) and (mild) central CH patients (Fig. 2), making the selection of a correct cutoff value for screening purposes difficult (65). Higher T4 cutoff values provide higher sensitivity at the cost of specificity (i.e., causing more false-positives).The choice between a fixed (absolute value) or floating (standard deviation of the daily mean) cutoff depends on size of the screened population, available resources and goal of screening. In case of a fixed cutoff value, laboratories should compare their methods in order to be able to use the same cutoff value. A floating cutoff value can be used between laboratories but needs a minimum number of samples per day in order to calculate a reliable daily mean and standard deviation.

In summary, screening for central CH needs a T4-based NBS approach with a carefully balanced cutoff value. Besides TBG-deficiency, other low T4 states that are frequently encountered in sick and preterm babies can be a cause of false-positive screening results. To differentiate other low T4 conditions from central CH (and to not miss primary CH in case of a blunted TSH response), adequate post-screening strategies (e.g., repeat testing) are necessary.

## Challenges of confirmatory testing after an abnormal screening result

In case of an abnormal screening result suggesting central CH, the newborn is referred to a pediatrician to confirm the diagnosis. After checking the medical history and performing a physical examination with specific attention for signs of congenital hypopituitarism, serum fT4 and TSH are measured (Fig. 1). The interpretation of confirmatory fT4 testing after referral is complex due to the physiological changes in thyroid hormone homeostasis in the neonatal period (66). Birth initiates a TSH surge, followed by marked increases in serum T4 and T3, peaking at 48–72 h postpartum (67). In the following weeks, thyroid hormone concentrations decline toward the adult reference interval (RI) (67). For correct interpretation, age-specific RIs are thus necessary (68). A Dutch study using the Cobas immunoassay (Roche Diagnostics, Germany) established neonatal RI for serum fT4 in 120 healthy newborns, finding a lower limit of 15.3 pmol/L at 2 weeks of age, which is significantly higher than the assay’s adult lower limit (12–22 pmol/L). These findings stress the importance of using a fT4 assay with a reliable neonatal RI. Using an incorrect (adult) RI can result in wrongly dismissing the diagnosis central CH, especially in mild (or masked, see below) cases.

To illustrate this problem, we conducted a small retrospective chart review from 2018 to 2023 to assess the number of neonates with borderline NBS results (*n* = 20) who were initially considered ‘normal’ after performing confirmatory thyroid function tests in the second week of life but would have been considered ‘abnormal’ based on the recently published age-specific RI (68). Our findings revealed that we had missed at least 10% of central CH cases based on the new RI, including a case of MPHD that mean while had presented with severe hypoglycemia due to combined growth hormone and ACTH deficiency (M R Garrelfs, N Zwaveling-Soonawala and A S P van Trotsenburg, unpublished observations).

Unfortunately, even optimal screening algorithms and post-screening strategies will continue to miss subgroups of patients, specifically in situations where central hypothyroidism evolves overtime or is masked by concurrent GHD (69). Van Iersel *et al.* studied thyroid function in children with presumed isolated congenital GHD between 2001–2011, and found that in 29 of 367 (7.9%) children, central CH was unmasked around the initiation of growth hormone treatment (69). Despite the relatively lenient T4 cutoff value used in the Dutch NBS program, the 29 children in this study were not detected (69).

In conclusion, despite advancements in confirmatory testing and the implementation of age-specific RI, challenges remain in accurately detecting and diagnosing central CH, particularly in neonates with concurrent conditions like GHD.

## Current screening practices

Countries or regions with a NBS program that currently allows detection of central CH are summarized in Table 1. In the United States of America (USA), 29 of the 51 NBS programs (57%) are able to detect central CH by performing either total T4-reflex TSH or simultaneous total T4 and TSH measurements (70, 71). Multiple states in the USA use floating total T4 cutoff values to perform the reflex TSH measurement. The cutoff is higher in the Netherlands (lowest 20%) compared to the USA (lowest 10%). A higher total T4 cutoff value will increase sensitivity at the cost of specificity, leading to more false-positive results. Reducing the number of false-positives while maintaining adequate sensitivity for detecting central CH was the main factor behind the evolution of the Dutch NBS program (see below) (27).

**Table 1:** Countries, regions and states that currently have the possibility to screen for central congenital hypothyroidism with their NBS program.

Country	Screening method	(f)T4 cutoffs
Italy (several regions) (33)	T4 + TSH simultaneously	Referral if T4 <40 nmol/L
Spain (several regions) (75)	T4 + TSH simultaneously	Referral if T4 <6 μg /dL
Malta (73)	T4 + TSH simultaneously	Referral if T4 <50 nmol/L
Japan (several regions) (52)	fT4 + TSH simultaneously	Referral if fT4 <0.5 ng/dL; second heel prick if FT4 <1.0 ng/dL, then referral if FT4 <1.0 ng/dL
Netherlands (27)	T4-reflex TSH-reflex TBG	Referral if T4 ≤−3 SD and TBG >40 nmol/L; if T4 <−1.6 SD, then TBG measurement; referral if T4/TBG ratio ≤17 in 1st and 2nd NBS result
United States of America (various regional/state programs) (70, 71)	Various approaches: T4 + TSH simultaneously, T4-reflex TSH and TSH-reflex T4	Various approaches
United States of America (Northwest Regional NBS Program) (70, 71)	T4 reflex TSH	Referral if T4 <10th percentile
Israel (32)	T4 reflex TSH	Reflex TSH if T4 <10th percentile, referral if TSH is elevated

Abbreviations: fT4, free thyroxine; TBG, thyroxine-binding globulin; TSH, thyroid-stimulating hormone; T4, total thyroxine; NA, not available.

Malta, and several regions in Spain and Italy, include total T4 testing in their NBS programs and use fixed cutoff values for further testing (72, 73, 74, 75).

In Japan, the Sapporo city region and Kanagawa prefecture stand out for their implementation of simultaneous TSH and fT4 measurements (52, 53, 76, 77). FT4 measurement seems to be a good alternative strategy to the Dutch three-step approach and calculation of the T4/TBG ratio to reduce the number of false-positives caused by TBG deficiency (54).

Interestingly, Israel employs a total T4-reflex TSH NBS program capable of detecting central CH that was introduced in 1978. However, children are only referred when an abnormal TSH value is encountered, thereby missing central CH cases. The earlier mentioned retrospective analysis of central CH patients by German *et al.* revealed that, of the 44 central CH patients that had undergone NBS between January 2008 and December 2021, only 13.6% had a total T4 value above the floating cutoff (lowest 10%) (32). This implies that 86.4% of patients could have been detected by NBS.

The most recent refinement of the Dutch NBS program was done in 2021, introducing a higher cutoff for TBG, and thereby further reducing the number of false-positive results due to (partial) TBG deficiency (27, 51). Nevertheless, even after this refinement, the positive predictive value is still only 21% for central CH (51). The false-positives are now mainly comprised of newborns with transient causes of a decreased T4 (e.g., NTI and borderline prematurity) and otherwise healthy newborns with a T4 in the lowest quintile of the normal distribution, as described above.

**Figure 3:**
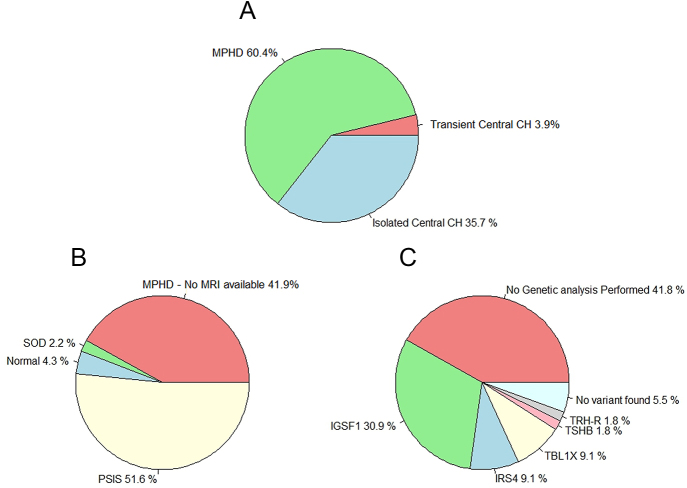
Results of 20 years (1995–2015) Dutch NBS for central congenital hypothyroidism. Pie chart delineating the results of 20 years (1995–2015) of NBS for central CH in the Netherlands. Total number of central CH cases detected by screening was 154, divided in 93 patients with MPHD, 55 patients with isolated central CH and six patients with transient central CH. (A) Division in isolated central CH, MPHD and transient central CH. (B) MRI results of MPHD patients. (C) Genetic results of isolated central CH cases. Abbreviations: CH, congenital hypothyroidism; MPHD, multiple pituitary hormone deficiencies; MRI, magnetic resonance imaging; PSIS, pituitary stalk interruption syndrome; SOD, septooptic dysplasia.

**Figure 4:**
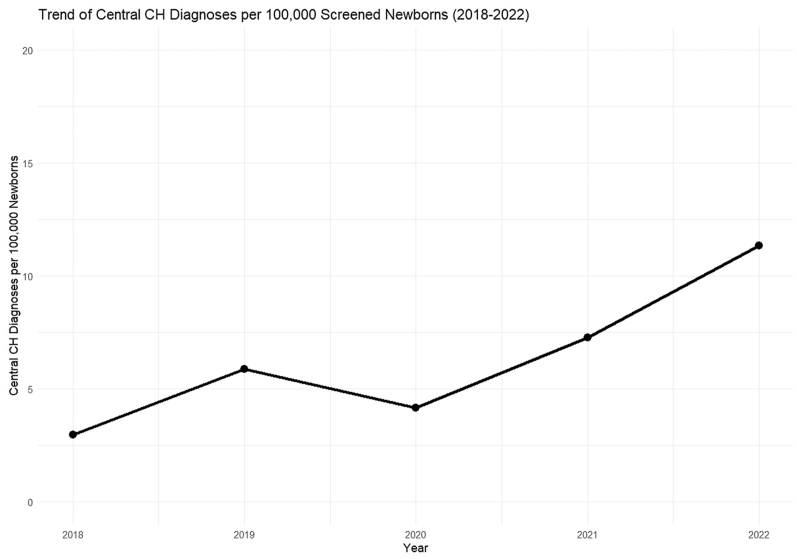
Trend of Dutch central congenital hypothyroidism diagnoses per 100,000 screened newborns (2018–2022).

The Dutch screening program for CH performs equally well at detecting primary CH as TSH-based methods (27, 49). The yield of the Dutch NBS program for central CH between 1995 and 2015 was reported by Naafs *et al.* in 2020 and is summarized in Fig. 3. Over 20 years, the total number of identified central CH cases was 154, divided in 93 (60.4%) patients with MPHD, 55 (35.7%) patients with isolated central CH and six (3.9%) patients with transient central CH. More recent data show an increase in the number of newborns diagnosed with central CH since 2021 (Fig. 4). This most likely results from recent improvements in interpreting confirmatory testing in the first two weeks of life due to new age-specific RIs for serum fT4, as described earlier (68).

In 2005, Lanting *et al.* evaluated the cost-effectiveness of the Dutch NBS strategy (T4-reflex TSH-reflex TBG) (49). Besides laboratory costs, their calculations included the costs of initial diagnostic evaluations after referral (i.e., one consultation with a general practitioner, one pediatric consultation and the initial diagnostic laboratory testing). Compared to other T4-based screening strategies, the addition of a TBG measurement increased the average costs to detect one patient only slightly (from $6209–6353 to $6851). The additional marginal costs, i.e. the extra costs required for each additional case detected when compared to the other methods, were calculated to be $11,206 (at the time of calculation, the exchange rate was 1 US dollar = 1 euro). Following the introduction of a higher TBG cutoff in 2021, costs have decreased due to a reduction in false-positive referrals (78). As TSH tests are more expensive than T4 tests, switching to a TSH-based NBS method would actually increase the average cost per case detected to $10,196 and miss central CH cases (49). Considering the lifetime healthcare costs and loss of productivity associated with untreated or delayed treatment of central CH/MPHD, screening for central CH is anticipated to be cost-effective.

In summary, while various countries have implemented NBS strategies for detecting central CH, these programs demonstrate significant differences in methodology, cutoff values and their ability to identify central CH cases.

## Future of screening for central CH

The most recent European Society for Pediatric Endocrinology guideline on CH recommends that adding total T4 or fT4 measurements to NBS programs should be considered in order to detect central CH (4). An increasing number of countries are now considering including this condition in their NBS programs (32, 72). However, adapting an existing NBS program can pose logistical challenges and may have financial implications.

When a T4-based screening method is chosen, either the measurement of TBG to calculate a T4/TBG ratio or replacing total T4 by direct measurement of fT4 could be incorporated to reduce the number of false-positives (54). As previously discussed, even when taking the effects of binding proteins into account, both total and free T4 remain imperfect biomarkers for central CH. New biomarkers or combinations of biomarkers may aid in differentiating central CH from other low T4 conditions.

Adding T3 and rT3 measurements is one of the possibilities. Measuring rT3 might reduce the number of false-positives due to NTI, although its diagnostic value needs further evaluation. A recent study looked at the feasibility of performing T3/rT3 measurements in dried blood spots with the goal of detecting monocarboxylate transporter 8 (MCT8) deficiency (79). MCT8 deficiency is yet another cause of low T4 levels in combination with normal TSH levels, that can potentially be picked up by T4-based NBS programs (although the biochemical footprint might resemble thyroid hormone resistance alpha) (80, 81). It was found that rT3 was significantly lower and the T3/rT3 ratio was higher in samples from the MCT8-deficient newborns compared to healthy controls (79). Premature newborns had low T3 and rT3 levels comparable to healthy controls, resulting in a low T3/rT3 ratio (79). Currently, there is no data on T3/rT3 concentrations in dried blood spots of central CH patients. Further research is needed to evaluate whether adding these measurements (and calculation of different ratios) to NBS programs can improve performance.

In recent years, genomic NBS has gained momentum due to factors such as increased processing speed and declining costs of next-generation sequencing (82, 83, 84, 85). Genomic NBS has the potential to identify a vast number of rare diseases quickly after birth and has already been successfully implemented in the screening for several conditions including cystic fibrosis, X-linked adrenoleukodystrophy and severe combined immunodeficiency (86). One of the major benefits of genomic screening in regards to central CH is that the assay is not influenced by binding proteins and performs equally well in healthy, sick and premature newborns. Despite its promise, genomic NBS still faces several hurdles, including the high incidence of non-disease-causing variants, correct variant interpretation in the absence of a phenotype and issues related to variable penetrance and expressivity of inherited disorders (87, 88). In cases where central CH is associated with MPHD, the yield of genetic testing is very low (<10–20% of cases), rendering genomic screening unsuitable for this condition at present (12, 13, 14, 15, 16, 17, 18). Genomic NBS can prove useful in detecting isolated central CH cases, as this is mainly a monogenic condition (6). Until the genetic causes of MPHD are better characterized (possibly by whole-genome sequencing), genetic evaluation can be used as a low-threshold second tier investigation mainly focused on the five known genes associated with isolated central CH (Fig. 1) (89).

Thyroid hormone is an important regulator of metabolism and affects most organ systems (90). If classical markers of hypothyroidism fail, the next logical step would be to look at the effects of thyroid hormones on metabolism (91). Since the 1990s, traditional ‘one test–one disorder’ methods have been replaced by tandem mass spectrometry (MS/MS), allowing the detection of multiple metabolites of varying chemical properties in a single assay (82). The combination of liquid chromatography (LC) and MS/MS further expanded the amount of metabolites analyzed and high-resolution mass spectrometers have made it possible to perform untargeted (‘shotgun’) metabolite analyses (metabolomics), detecting thousands of non-specific features in a single sample (82). In multifactorial conditions such as MPHD, the metabolome (or metabolic signature) might provide a closer correlation to the phenotype than the genome.

The metabolome was found to be relatively stable in dried blood spots, even after storage for several years, making metabolomics suitable for use in NBS programs (92). Metabolomics (including its subfield lipidomics) can be combined with other ‘omics’ analyses such as proteomics, epigenomics and transcriptomics (82, 93). These ‘multi-omics’ approaches provide great opportunities for biomarker discovery and feature selection, especially when used in synergy with sophisticated bioinformatics pipelines (94, 95, 96, 97).

Due to the enormous amount of data generated by omics analyses, innovative post-analytical methods are necessary for streamlining interpretation (97). Machine learning and artificial intelligence provide a great opportunity to improve the performance of NBS programs (93, 95, 98, 99). New ‘omics’ features can be added to classifier algorithms to improve the performance of existing NBS programs, and in the future, ‘multi-omics profiles or signatures’ could have the potential to replace the classical way of screening (82, 93, 100).

## Concluding remarks

We reviewed the past, present and future of NBS for central CH. There is increasing evidence for a positive effect of early treatment of this condition on neurodevelopment. In addition, central CH is currently the only pituitary hormone deficiency suitable for NBS and therefore has the potential to improve early diagnosis of the potentially fatal condition MPHD. These factors stress the importance of including central CH in NBS programs around the world. We elaborated on the pitfalls of NBS for central CH including clinical condition of the patient, gestational age, binding proteins and the individual setpoint of the HPT axis. Finally, possible future directions of NBS for central CH were discussed.

As central CH is a relatively rare condition, international collaboration is needed to investigate new screening strategies and quantify the actual (cost-)benefit of early detection of central CH.

## Declaration of interest

The authors declare that there is no conflict of interest that could be perceived as prejudicing the impartiality of the work.

## Funding

This work did not receive any specific grant from any funding agency in the public, commercial or not-for-profit sector.

## Author contribution statement

M R Garrelfs performed data acquisition, designed the tables and figures and drafted the article. A Boelen, C F Mooij, A S P van Trotsenburg and N Zwaveling-Soonawala reviewed and edited the manuscript.

## Ethics

All procedures were performed in accordance with the Declaration of Helsinki.
